# Acute Abdomen Resulting from Concurrent Thrombosis of Celiac Trunk and Superior Mesenteric Artery

**DOI:** 10.1155/2014/142701

**Published:** 2014-09-23

**Authors:** Savaş Bayrak, Hasan Bektas, Yigit Duzkoylu, Ayhan Guneyi, Ekrem Cakar

**Affiliations:** Istanbul Training and Research Hospital, General Surgery Clinic, Fatih, 34098 Istanbul, Turkey

## Abstract

Mesenteric ischemia is one of the most mortal diseases of the gastrointestinal system causing acute abdomen. In most of the patients, the etiological factor is the obstructive embolism or thrombosis of superior mesenteric artery. In the literature, there have been reports regarding also celiac trunk occlusion in rare situations. The gold standard treatment relies on early diagnosis. The originality of our report relies on the concurrent obstruction of both vascular structures.

## 1. Introduction

Mesenteric ischemia accounts for 2% of cases with gastrointestinal diseases causing acute abdomen [1]. Although it is rare, mortality rates of acute mesenteric ischemia can reach up to 40–70% [2]. In 70–80% of the patients, the etiological factor is the obstructive embolism or thrombosis of superior mesenteric artery (SMA) [2]. In the literature, there have been reports regarding also celiac trunk (CT) occlusion in rare situations. The originality of our report relies on the concurrent obstruction of both vascular structures.

## 2. Case Report

**Figure 1:**
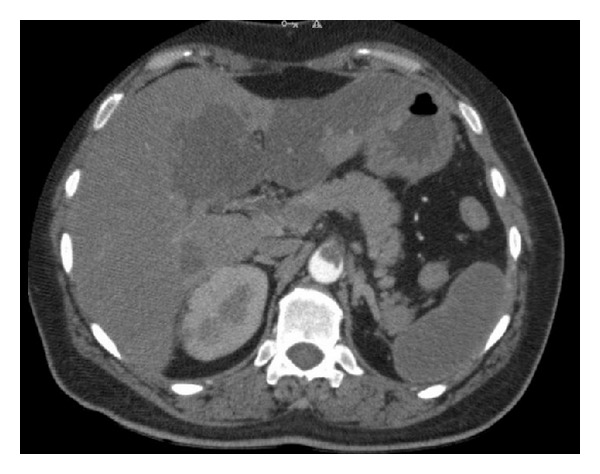
Celiac trunk is obstructed 100% and SMA is obstructed 80%; there are hypodense areas on the liver and the spleen.

**Figure 2:**
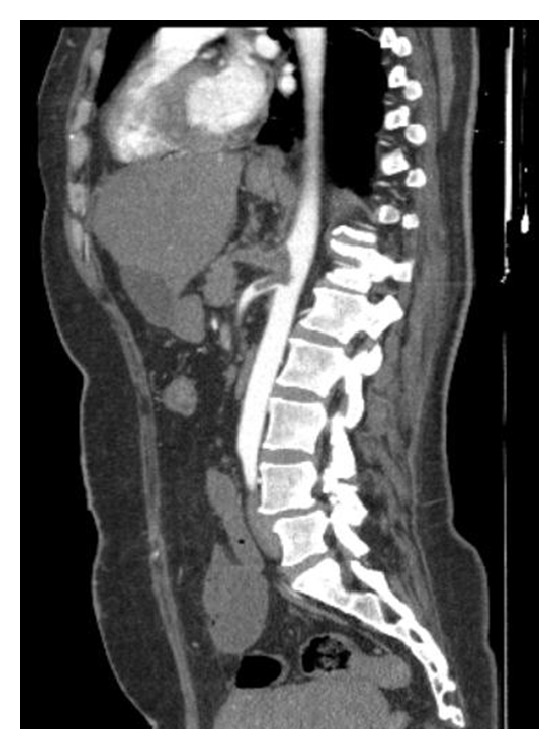
Sagittal image of solid organs with hypodense areas.

A 44-year-old woman was referred to emergency department with abdominal pain lasting for 1 week. She did not have a particular medical history except a nephrectomy performed 10 years ago because of renal calculi. In her physical examination, there were signs of peritoneal irritation and diffuse abdominal tenderness. Her blood pressure was 100/80 mm/Hg, body temperature was 38.1°C, heart rate was 115/min, LDH was 485 IU/L, PT-INR was 1.09, and level of glucose was found to be 193 mg/dL. In computerized tomographic angiography, 100% of CT and 80% of SMA were obstructed; there were hypodense areas on the left and right anterior segments of the liver, and the spleen was also hypodense; gallbladder was normal and intestines were edematous (Figures 1 and 2). The patient was interned to the operation room with symptoms of acute abdomen. There was necrosis in the segment beginning from the 90th cm from Treitz to the 30th cm before ileocecal valve and the gallbladder was also necrosed. Gangrenous parts of jejunum and ileum were resected, and the operation was completed with end jejunostomy and mucous fistula, including cholecystectomy. There were not any macroscopic pathological findings on either liver or spleen, so we did not perform anything on them. The patient was followed up in intensive care unit, and total parenteral nutrition was administered. Consultation was made with cardiology, cardiovascular surgery, and hematology departments. Echocardiography was normal. Serum levels of protein C, protein S, and antithrombin III were found to be normal. Oral intake was started on the 7th postoperative day. On the postoperative 8th day, after detecting infectious findings (WBC: 18000, sedimentation rate: 34 mm/h, CRP: 12 mg/L), consultation was made with infectious diseases physician and antibiotherapy was changed to piperacillin-tazobactam with metronidazole for 7 days. After detecting high urea (34 mg/dL) and creatinine (2.8 mg/dL), consultation was made with nephrology physician and the patient was treated with dialysis for 3 times in 7 days. Patient was discharged from the hospital on the 29th day with stable vital signs and normal blood and radiological findings.

She was reoperated on in the 6th month for closure of ostomies. An end-to-end full layer anastomosis was performed between jejunum and ileum segments. On the postoperative 4th day, she was reoperated on after detecting bile drainage. In the operation, there was found to be a leakage in the anastomosis and jejunostomy with mucous fistula was performed. In the intensive care unit, she died with disseminated intravascular coagulation on the postoperative 3rd day.

## 3. Discussion

### 3.1. Epidemiology

SMA and CT are the main sources that supply blood to the gastrointestinal system. In the autopsy series, over 50% of stenosis has been reported in at least one mesenteric artery, in 6–10% of the population [3]. While stenosis of SMA or CT is more than 50% in asymptomatic individuals, the rate is 27% in patients after performing arteriography [4]. Among factors that enable arterial thrombosis, there are atherosclerosis, myocardial infarction, congestive heart failure, coagulation anomalies, malignancy, and collagen tissue diseases [3].

### 3.2. Diagnosis

Angiography is the golden standard technique in diagnosis [5]. In mesenteric ischemia secondary to the thrombosis of CT, nonspecific clinical and laboratory findings cause delay in diagnosis and treatment [2]. Because of these factors, mortality of CT thrombosis in hospital is still 59–93% today [6]. Mesenteric ischemia results from arterial embolism in 50%, arterial thrombosis in 20%, nonocclusive mesenteric ischemia in 20%, and venous thrombosis in 10% of the patients [7]. In these vascular pathologies, SMA is the most common affected artery. Rarely, CT is occluded. Our aim is to report a case with obstruction in both of these arteries. In our case, while 80% of SMA was occluded, CT was occluded completely. Clinical suspicion is the mainstay of early diagnosis. Laboratory tests are nonspecific. Abdominal ultrasound examination can be helpful in diagnosis. Duplex ultrasonography can visualize SMA and CT [3]. In acute mesenteric ischemia, sensitivity of computerized arteriography is 100% and specificity is 89% [8]. Conventional selective angiography is the golden standard diagnostic tool [6]. It can be used also for treatment.

### 3.3. Treatment

After the diagnosis is certain, treatment options depend on many factors. The level of occlusion, collateral vasculature, and clinical state of the patient are among them. Surgery is the only option for patients with intestinal infarction and signs of acute abdomen and peritoneal irritation may give a hint for infarction. In diagnosed patients without these signs, endovascular interventions can be performed with low rates of complication and mortality [9]. Mesenteric bypass surgery is another treatment option in patients without intestinal gangrene and signs of acute abdomen. In a study that had evaluated both treatment modalities, rates of 3-year survival were similar but complication rates were higher in the group treated with mesenteric vascular bypass surgery. At the end of 3rd year, recurrence rates with clinical importance were found to be significantly higher for balloon angioplasty [8]. The fact that our patient had signs of acute abdomen and peritoneal irritation prevented us from performing these treatment modalities. It is of great importance to keep in mind the classical findings of chronic mesenteric ischemia, such as postprandial abdominal pain, weight loss, alteration in bowel functions, and “food fear” [10], to assess patients and advance from these treatment options before the development of end-organ damage. An appropriate treatment modality can be chosen for intestinal infarction with an imaging technique that is performed on the right time, so the rates of morbidity and mortality can be decreased. In our case, it puts us on the spot not to be able to evaluate this process.

An algorithm is essential for early diagnosis and treatment. In the literature, there are examples of attempts for forming an algorithm. In one of these studies, patients were divided into two groups. In the first group, the patients were unstable with classical signs of mesenteric ischemia such as abdominal pain out of proportion and tomography findings showing arterial occlusion. Endovascular treatment with the evaluation of intestinal ischemia in the operation room is advised for this patient group. In the other group, the patients were with nonspecific symptoms, resulting from venous thrombosis and nonocclusive mesenteric ischemia; bowel rest, regular physical examination, treatment of underlying disease and inflammatory process, and exploratory laparotomy for the treatment of vascular deficiency are advised [7]. Although an algorithm for treatment has been stated, a complete one for diagnosis and treatment has not been approved for clinical practice yet.

## 4. Conclusions

It is of great importance to be aware of clinical findings of acute and chronic mesenteric ischemia because of the worth of early diagnosis. With early diagnosis, there may be a chance for vascular surgical modalities and endovascular interventions. After the development of end-organ damage, the morbidity and mortality of any procedure can reach high rates.

At this point, the most important role is for the clinician. Clinical suspicion with early diagnosis has utmost importance for increasing survival rates. We aimed to emphasize the vital importance of an experienced surgical team, intensive care unit, the capability for endovascular interventions, and advanced imaging modalities in coping with such mortal entity.
